# Antimicrobial Resistance Patterns and Predictors in *Helicobacter pylori* Infection: A Real-World Cohort Study

**DOI:** 10.3390/microorganisms14051129

**Published:** 2026-05-16

**Authors:** Sergiu Dorin Matei, Ramona Nicoleta Suciu, Tiberia Ilias, Grațiela Aneta Avram, Corina Suteu, Laura Ioana Bondar, Cristian Hocopan, Carmen Pantis, Roland Fazakas, Ovidiu Frățilă

**Affiliations:** 1 Doctoral School of Biomedical Sciences, Faculty of Medicine and Pharmacy, University of Oradea, 410087 Oradea, Romaniabondar.lauraioana@student.uoradea.ro (L.I.B.);; 2 Department of Medical Disciplines, Faculty of Medicine and Pharmacy, University of Oradea, 410073 Oradea, Romania; 3 Department of Psycho-Neuroscience and Recovery, Faculty of Medicine and Pharmacy, University of Oradea, 410073 Oradea, Romania; 4 Department of Biology and Life Sciences, Faculty of Medicine, “Vasile Goldiș” Western University of Arad, 310025 Arad, Romania; fazakas.roland@uvvg.ro; 5 Department of Surgical Disciplines, Faculty of Medicine and Pharmacy, University of Oradea, 410073 Oradea, Romania; carmen.pantis@didactic.uoradea.ro; 6 Multidisciplinary Doctoral School, “Vasile Goldiș” Western University of Arad, 310025 Arad, Romania

**Keywords:** antimicrobial stewardship, anti-bacterial agents, clarithromycin, drug resistance, bacterial, *Helicobacter pylori*, multidrug resistance, treatment failure

## Abstract

Rising antimicrobial resistance has reduced the effectiveness of empirical eradication regimens for *Helicobacter pylori* (*H. pylori*) infection, particularly those containing clarithromycin. Local resistance surveillance and identification of clinical predictors of resistance are essential to guide treatment strategies. This study evaluated antimicrobial resistance patterns and clinical determinants of resistance in a real-world tertiary-care cohort. A retrospective observational study was performed, which included 352 adult patients with confirmed *H. pylori* infection managed between November 2022 and November 2025. Of these, 168 patients underwent culture and antibiotic susceptibility testing, while 184 received empirical therapy. Resistance rates were calculated according to the number of isolates tested for each antimicrobial agent (available-case analysis). Multivariable logistic regression analysis was used to identify independent predictors of resistance. Among susceptibility-tested patients, resistance to at least one antimicrobial agent was detected in 44.6%. Clarithromycin resistance was most frequent (42.5%), followed by metronidazole (36.4%) and levofloxacin (14.0%), whereas amoxicillin resistance remained low (2.4%). Multidrug resistance (MDR) based on available susceptibility data was observed in 12.5% of cases, most commonly involving dual clarithromycin–metronidazole resistance. Prior eradication therapy was independently associated with resistance (adjusted Odds Ratio aOR 2.41; 95% Confidence Interval CI 1.29–4.51; *p* = 0.006), while demographic factors were not. Clarithromycin resistance substantially exceeds recommended thresholds for empirical triple therapy in this setting. Prior eradication therapy is the principal predictor of resistance, supporting resistance-informed and stewardship-oriented management strategies.

## 1. Introduction

*Helicobacter pylori* (*H. pylori*) is a Gram-negative, microaerophilic bacterium that chronically colonizes the gastric mucosa and infects approximately half of the global population [1,2,3]. Persistent infection is associated with chronic gastritis, peptic ulcer disease, mucosa-associated lymphoid tissue lymphoma, and gastric adenocarcinoma, and it is classified as a class I carcinogen by the World Health Organization [4,5,6,7]. Effective eradication of *H. pylori* therefore remains a major objective in gastroenterology and infection prevention strategies worldwide.

Conventional first-line eradication regimens have historically relied on proton pump inhibitor (PPI)-based triple therapy combining amoxicillin and clarithromycin, with or without metronidazole [8,9]. However, eradication success rates have declined substantially over the past two decades, largely due to the global rise in antimicrobial resistance [8,10,11,12]. Clarithromycin resistance is widely recognized as the principal determinant of treatment failure, and when local resistance rates exceed 15–20%, the effectiveness of empirical clarithromycin-based triple therapy drops below acceptable levels [13,14]. Consequently, international guidelines now recommend avoiding clarithromycin-containing empirical regimens in high-resistance settings and favor bismuth-based quadruple therapy or susceptibility-guided approaches [13,15].

Despite these recommendations, empirical therapy continues to be widely used in routine practice, particularly in centers where culture and antibiotic susceptibility testing are not universally available [16,17,18,19]. Moreover, antimicrobial resistance patterns vary substantially across regions, reflecting differences in antibiotic consumption, healthcare structures, and prescribing practices [20,21,22,23]. Continuous local surveillance is therefore essential to ensure that empirical treatment strategies remain appropriate and effective.

Although increasing resistance to clarithromycin and metronidazole has been documented across Europe and globally important gaps remain. Real-world data from Eastern European tertiary-care populations are relatively limited, especially in cohorts including both susceptibility-tested and empirically treated patients. Furthermore, while prior eradication therapy is considered a major risk factor for secondary resistance, its independent contribution relative to demographic and clinical factors is not consistently quantified in routine clinical settings. Few studies integrate updated local resistance prevalence with multivariable modeling to identify clinically actionable predictors that could support individualized, resistance-informed management where universal susceptibility testing is not feasible [20,24,25,26].

Given the clinical and public health implications of rising resistance, there is a need for contemporary regional data that combine microbiological surveillance with evaluation of patient-level determinants of resistance. Such information is essential to inform rational empirical therapy selection, minimize treatment failure, and strengthen antimicrobial stewardship efforts.

The present study was therefore designed to evaluate antimicrobial resistance patterns in *H. pylori* infection within a real-world tertiary-care cohort and to identify clinical predictors associated with resistance. By providing updated local data and examining the determinants of resistance in routine practice, this study aims to support more effective and stewardship-oriented management strategies in high-resistance settings.

## 2. Materials and Methods

### 2.1. Study Design and Setting

This retrospective observational cohort study was conducted at the Internal Medicine Department of the Oradea County Emergency Hospital, Oradea, Romania. The hospital is a tertiary referral center providing inpatient and outpatient medical services to a large regional population.

The study included adult patients diagnosed with *H. pylori* infection who were hospitalized or evaluated in the outpatient department between 1 November 2022 and 1 November 2025. Clinical, endoscopic, microbiological, and treatment data were collected from electronic medical records and institutional databases.

### 2.2. Study Population and Eligibility Criteria

A total of 384 adult patients with a documented diagnosis of *H. pylori* infection were initially identified through systematic review of the hospital’s electronic medical records system and archived paper medical files during the study period.

Inclusion criteria were:

• Age ≥18 years;
• Confirmed diagnosis of *H. pylori* infection established by rapid urease test (RUT) and/or stool antigen testing;
• Availability of sufficient clinical and treatment information for analysis.
• Patients were excluded if they:
• Had incomplete or missing essential clinical or microbiological data;
• Lacked documented confirmation of *H. pylori* infection;
• Had insufficient information regarding prior eradication therapy when applicable.

Of the 384 identified patients, 32 were excluded due to incomplete or missing essential clinical or microbiological data (*n* = 18), lack of documented confirmation of *H. pylori* infection (*n* = 9), or insufficient information regarding prior eradication therapy (*n* = 5).

**Figure 1:**
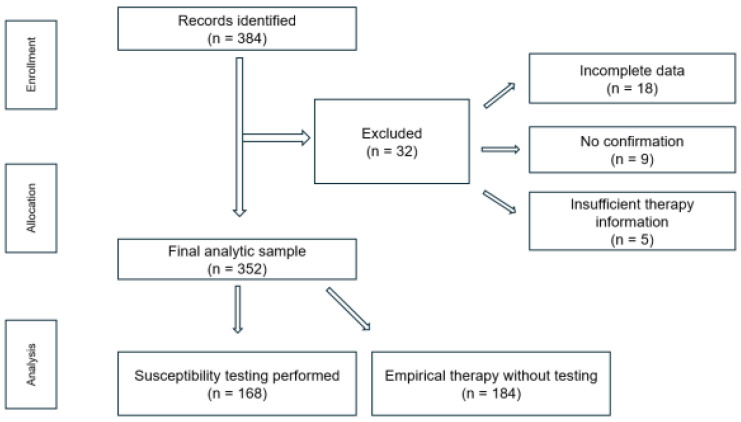
Flow diagram of participant recruitment and inclusion in the study.

The final analytic sample consisted of 352 consecutive adult patients (≥18 years), who were included in all subsequent analyses (Figure 1).

### 2.3. Diagnostic Confirmation of H. pylori Infection

All included patients underwent upper gastrointestinal endoscopy during hospitalization. The procedure was performed using a high-definition video endoscopy system (Olympus Medical Systems, Tokyo, Japan). Infection was confirmed by RUT performed on gastric biopsy specimens obtained during the procedure using a commercially available kit (HelicotecUT^®^ Plus, Strong Biotech, Taipei, Taiwan). Biopsy samples were typically collected from the gastric antrum and processed according to the manufacturer’s instructions.

In a subset of patients, stool antigen testing had been performed prior to hospitalization as part of outpatient evaluation using a commercial enzyme immunoassay kit (CerTest *H. pylori*, CerTest Biotec, Zaragoza, Spain). These results were recorded in the medical files and included in the analysis; however, biopsy-based confirmation was available for all patients included in the final cohort.

A positive RUT result was considered sufficient to confirm active *H. pylori* infection for study inclusion.

### 2.4. Group Allocation and Treatment Approach

Patients were allocated into two groups based on whether antibiotic susceptibility testing was performed prior to initiation of eradication therapy.

• Group 1: Susceptibility-guided therapy (*n* = 168). Patients in this group underwent upper gastrointestinal endoscopy with gastric biopsy sampling followed by culture and antibiotic susceptibility testing. Eradication therapy was subsequently selected according to the individual antimicrobial susceptibility profile when available.
• Group 2: Empirical therapy without susceptibility testing (*n *= 184). Patients in this group received empirical eradication therapy without prior culture or susceptibility testing. Treatment regimens were prescribed according to routine clinical practice and prevailing guideline recommendations at the time of management.

Group allocation was not randomized but reflected real-world clinical decision-making and resource availability within the study center. The decision to perform susceptibility testing was based on physician judgment, clinical presentation, prior eradication history, and logistical considerations.

### 2.5. Culture and Antibiotic Susceptibility Testing

In patients allocated to the susceptibility-guided group, gastric biopsy specimens obtained during upper gastrointestinal endoscopy were submitted for microbiological analysis.

Biopsy samples were cultured on Columbia agar supplemented with 5% sheep blood (bioMérieux, Marcy-l’Étoile, France) and incubated under microaerophilic conditions generated using CampyGen™ gas-generating sachets (Oxoid Ltd., Basingstoke, UK) at 37 °C for up to 5–7 days. Identification of *H. pylori* isolates was based on colony morphology and conventional biochemical characteristics.

Antibiotic susceptibility testing was performed by determining minimum inhibitory concentrations (MICs) using E-test strips (bioMérieux, Marcy-l’Étoile, France). Interpretation of susceptibility or resistance was performed according to the European Committee on Antimicrobial Susceptibility Testing (EUCAST) clinical breakpoints (version 13.0, Växjö, Sweden, 2023). Quality control was performed using reference strains in accordance with standard laboratory procedures.

The antibiotics tested included amoxicillin, clarithromycin, metronidazole, levofloxacin, moxifloxacin, and rifampicin. Due to technical and laboratory constraints, not all isolates were tested against every antimicrobial agent.

Resistance was defined according to the applicable breakpoint thresholds, and multidrug resistance (MDR) was defined as resistance to two or more antimicrobial classes, in accordance with commonly accepted definitions.

### 2.6. Clinical and Demographic Variables

Clinical and demographic data were extracted from electronic medical records and archived paper files using a standardized data collection form.

Demographic variables included age (recorded as a continuous variable), sex (male/female), and place of residence (urban vs. rural).

Clinical variables included presenting symptoms (e.g., epigastric pain, diffuse abdominal pain, melena, nausea, flatulence, hematemesis, loss of appetite, rectal bleeding, vomiting, and heartburn), history of prior *H. pylori* eradication therapy, and details of previously administered treatment regimens when available.

Endoscopic findings were recorded for patients who underwent upper gastrointestinal endoscopy and included gastritis subtypes (erythematous, erosive, edematous, atrophic), ulcerative lesions (gastric and duodenal ulcers), esophageal ulcerations, and presence of gastric malignancy.

For patients in the susceptibility-guided group, microbiological variables included antibiotic susceptibility results for amoxicillin, clarithromycin, metronidazole, levofloxacin, moxifloxacin, and rifampicin.

All variables were recorded as documented in the medical records without modification or imputation. Missing data were not imputed and were handled using an available-case approach in the statistical analysis, with denominators adjusted according to data availability.

### 2.7. Outcome Measures

The primary outcome of the study was the presence of antimicrobial resistance, defined as resistance to at least one tested antibiotic among patients who underwent susceptibility testing.

Secondary outcomes included:

• The prevalence of resistance to individual antibiotics (amoxicillin, clarithromycin, metronidazole, levofloxacin, moxifloxacin, and rifampicin);
• The prevalence of MDR, defined as resistance to two or more antimicrobial classes;
• The association between prior *H. pylori* eradication therapy and the occurrence of antimicrobial resistance;
• Identification of independent clinical predictors of resistance using multivariable logistic regression analysis.

### 2.8. Assessment of Eradication Outcomes

Eradication outcomes were assessed in patients with available post-treatment confirmation of *H. pylori* infection. Test-of-cure was performed using stool antigen testing or urea breath testing at least four weeks after completion of eradication therapy, in accordance with guideline recommendations.

Eradication was defined as a negative test result, indicating successful clearance of infection, while a positive result was considered treatment failure.

Due to the retrospective design and variability in follow-up practices, eradication outcomes were not available for all patients. Therefore, analyses of eradication rates were performed using an available-case approach, including only patients with documented post-treatment testing.

### 2.9. Statistical Analysis

Statistical analyses were performed using IBM SPSS software (version 26.0; IBM Corp., Armonk, NY, USA).

Continuous variables were expressed as mean ± standard deviation (SD), while categorical variables were presented as frequencies and percentages. Normality of continuous variables was assessed using the Shapiro–Wilk test, supplemented by visual inspection of histograms and Q–Q plots.

Comparisons between groups were performed using the independent-samples t-test for normally distributed continuous variables and the Mann–Whitney U test for non-normally distributed variables, as appropriate. Categorical variables were compared using the chi-square (χ^2^) test or Fisher’s exact test when expected cell counts were less than five.

Antibiotic resistance rates were calculated according to the number of isolates tested for each antimicrobial agent. MDR was defined as resistance to two or more antimicrobial classes.

To identify independent predictors of antimicrobial resistance, multivariable logistic regression analysis was performed among patients who underwent susceptibility testing. Resistance to at least one antimicrobial agent was defined as the dependent variable. Independent variables included age (continuous), sex, place of residence (urban vs. rural), and history of prior *H. pylori* eradication therapy.

Adjusted odds ratios (aORs) with 95% confidence intervals (CIs) were calculated. Model discrimination was assessed using the area under the receiver operating characteristic curve (AUC), and calibration was evaluated using the Hosmer–Lemeshow goodness-of-fit test. The events-per-variable (EPV) ratio was calculated as the number of outcome events divided by the number of independent variables included in the model. In the present analysis, 75 events and 4 predictor variables yielded an EPV of 18.8, which exceeds the commonly recommended minimum threshold of 10 events per variable for reliable logistic regression models.

All statistical tests were two-tailed, and a *p*-value < 0.05 was considered statistically significant.

Due to laboratory and technical constraints, not all isolates were tested against every antimicrobial agent. Missing susceptibility data were not imputed. Analyses of antibiotic-specific resistance rates were performed using an available-case approach, with denominators corresponding to the number of isolates tested for each individual antibiotic (ranging from 120 to 164 isolates). This variability in testing coverage should be considered when interpreting resistance prevalence estimates. For analyses involving the composite outcome of resistance to at least one antimicrobial agent, only patients with at least one valid susceptibility result were included.

Comparisons of eradication rates between treatment groups (susceptibility-guided versus empirical therapy) and according to antimicrobial resistance status were performed using the chi-square (χ^2^) test or Fisher’s exact test, as appropriate, in the subset of patients with available follow-up data.

### 2.10. Ethical Considerations

The study protocol was approved by the Research Ethics Subcommittee of the University of Oradea (Approval No. 86/31.03.2026) and by the Ethics Committee of the Oradea County Emergency Clinical Hospital, Romania (Approval No. 33050/06.11.2025). All procedures were conducted in accordance with the ethical standards of the Declaration of Helsinki and its later amendments.

Given the retrospective nature of the study and the use of anonymized clinical data extracted from medical records, the requirement for informed consent was waived by the Ethics Committee.

All patient data were handled confidentially and analyzed in anonymized form to ensure compliance with institutional and national data protection regulations.

### 2.11. Hypotheses of the Study

Based on previous evidence demonstrating increasing antimicrobial resistance in *H. pylori* infection, declining effectiveness of empirical eradication regimens, and the potential impact of prior antibiotic exposure on resistance development, the present study examined the following hypotheses:

1. Prior *H. pylori* eradication therapy is independently associated with an increased likelihood of antimicrobial resistance, particularly resistance to clarithromycin and MDR patterns.
2. A substantial prevalence of antimicrobial resistance exists in the study population, with clarithromycin and metronidazole resistance representing the most frequently observed resistance patterns.
3. MDR occurs more frequently among patients with a history of prior eradication therapy compared with treatment-naïve patients.
4. Demographic factors, including age, sex, and place of residence, are not independent predictors of antimicrobial resistance after multivariable adjustment.
5. The prevalence of clarithromycin resistance in the study population exceeds the 15–20% threshold recommended by international guidelines, thereby questioning the appropriateness of empirical clarithromycin-based triple therapy in this setting.

## 3. Results

### 3.1. General Characteristics of the Study Population

A total of 352 patients with confirmed *H. pylori* infection were included in the analysis. Among them, 168 patients underwent antibiotic susceptibility testing (study group), while 184 received empirical eradication therapy without prior susceptibility testing (control group).

The mean age of the overall cohort was 51.8 ± 15.6 years. Patients in the study group were slightly younger than those in the control group (49.6 ± 15.2 vs. 53.9 ± 15.7 years, *p* = 0.012).

Male sex was moderately more frequent in the study group compared with the control group (58.3% vs. 50.0%, *p* = 0.118). The distribution of place of residence (urban origin) was comparable between groups (61.9% vs. 59.2%, *p* = 0.642).

Baseline demographic characteristics were comparable between groups, except for age (Table 1).

Baseline demographic characteristics were broadly comparable between groups, with the exception of age, which was slightly lower in the study group. Although this difference reached statistical significance, the absolute difference in mean age was modest. Importantly, age was not associated with antimicrobial resistance in multivariable analysis. Furthermore, apart from age and prior eradication therapy, no statistically significant differences were observed between groups across clinical presentation and endoscopic findings, indicating overall comparability of baseline characteristics.

### 3.2. Clinical Presentation

The distribution of presenting symptoms was generally comparable between the study and control groups. Epigastric pain was the most common symptom overall, reported by more than 90% of patients in both groups. Diffuse abdominal pain was observed in 64.3% of patients in the study group and 58.7% in the control group (*p* = 0.284).

Melena was reported in 11.3% of patients undergoing susceptibility testing compared with 6.0% in the empirical treatment group (*p* = 0.082). Other symptoms—including nausea, flatulence, hematemesis, loss of appetite, rectal bleeding, vomiting, and heartburn—were similarly distributed between groups, with no statistically significant differences observed.

No statistically significant differences were observed in the distribution of presenting symptoms between groups, suggesting a broadly similar clinical presentation at baseline (Table 2).

### 3.3. Diagnostic Methods

All 352 included patients underwent upper gastrointestinal endoscopy with RUT during hospitalization, which served as the definitive diagnostic confirmation of *H. pylori* infection for study inclusion.

In a subset of patients, stool antigen testing had been performed prior to hospitalization as part of outpatient evaluation. These results were documented in the medical records and were similarly distributed between the study and control groups (33 [19.6%] vs. 39 [21.2%], *p* = 0.764).

### 3.4. History of Previous H. pylori Eradication Therapy

A history of prior *H. pylori* eradication therapy was more frequently observed in the study group than in the control group (22.6% vs. 11.4%, *p* = 0.006).

Among previously treated patients in the study group, the most common regimen consisted of PPI-based triple therapy combining amoxicillin and clarithromycin. A smaller proportion of patients had received second-line regimens including levofloxacin-based combinations. Similar patterns were observed in the control group, although prior therapy was less frequent overall.

A higher proportion of patients in the study group had a history of prior eradication therapy (Table 3).

### 3.5. Biopsy Findings

All 352 patients underwent upper gastrointestinal endoscopy with biopsy sampling during hospitalization. The gastric antrum was the most frequently sampled site in both groups, with biopsies obtained in 139/168 (82.7%) patients in the study group and 150/184 (81.5%) in the control group. In some patients, additional or alternative biopsy sites were selected based on endoscopic findings and clinical judgment. However, the distribution of biopsy sites did not differ significantly between groups (*p* = 0.874), suggesting that variability in biopsy site selection was unlikely to have introduced systematic bias in comparative analyses.

Biopsies from the gastric body were obtained in 20/168 (11.9%) and 25/184 (13.6%) patients, respectively, while duodenal bulb biopsies were performed less frequently (5.4% vs. 4.9%).

### 3.6. Endoscopic Findings

Endoscopic findings were heterogeneous across the cohort. Erythematous gastritis was the most frequently observed finding in both groups (46.4% in the study group vs. 48.9% in the control group; *p* = 0.648). Erosive gastritis was identified in 18.5% and 16.3% of patients, respectively.

Atrophic gastritis was observed in a minority of patients without significant intergroup differences (8.9% vs. 10.3%, *p* = 0.671). Edematous gastritis and cases with no visible macroscopic lesion were infrequent and similarly distributed between groups.

Ulcerative disease was present in both cohorts. Gastric ulcers were identified in 9.5% of patients in the study group and 6.5% in the control group (*p* = 0.284), while duodenal ulcers were observed in 11.3% and 8.7% of patients, respectively (*p* = 0.411). Esophageal ulcerations were uncommon. No statistically significant differences in the overall distribution of endoscopic findings were detected between groups (χ^2^ test, *p* = 0.572).

No cases of gastric malignancy were identified during the study period (Table 4).

### 3.7. Antibiotic Susceptibility Patterns in the Study Group

Antibiotic susceptibility testing was successfully performed in 168 patients in the study group. Due to technical and laboratory constraints, not all isolates were tested against every antimicrobial agent; therefore, resistance rates are reported using an available-case approach, according to the number of isolates tested for each antibiotic. The proportion of isolates tested varied across antibiotics (120–164 isolates), which may influence the comparability and precision of resistance rates between antimicrobial agents.

Clarithromycin resistance was the most frequently observed resistance pattern, detected in 68 of 160 tested isolates (42.5%). Metronidazole resistance was identified in 59 of 162 isolates (36.4%), while levofloxacin resistance was observed in 21 of 150 isolates (14.0%). Amoxicillin resistance remained low, detected in 4 of 164 tested isolates (2.4%). Resistance to rifampicin was rare (2 of 120 isolates, 1.7%).

Among the 168 patients undergoing susceptibility testing, and based on available susceptibility data for each isolate, resistance to at least one antimicrobial agent was detected in 75 patients (44.6%). Single-drug resistance was identified in 54 patients (32.1%), while MDR (resistance to ≥2 antimicrobial classes) was observed in 21 patients (12.5%).

Among isolates with available susceptibility profiles, the most frequently observed MDR pattern was dual resistance to clarithromycin and metronidazole. No additional resistance combinations were observed among isolates with sufficiently complete susceptibility data. However, interpretation of MDR distribution should be made cautiously because not all isolates underwent susceptibility testing against all antimicrobial agents. Consequently, incomplete testing coverage may have limited the identification of less frequent or alternative resistance combinations and may affect the completeness of MDR characterization.

Because composite resistance categories were derived from available susceptibility data rather than complete antibiotic profiles for all isolates, the reported MDR patterns should be considered exploratory observations rather than definitive estimates of the full resistance spectrum within the study population.

These findings indicate a high prevalence of clarithromycin resistance in the study population, while the observed MDR patterns should be interpreted cautiously because of incomplete susceptibility testing across antimicrobial agents (Table 5 and Table 6).

A descriptive breakdown of the observed dual resistance patterns based on available susceptibility data is presented in Table 7. Among isolates with sufficiently complete susceptibility profiles, dual resistance to clarithromycin and metronidazole was the predominant MDR pattern identified. However, because susceptibility testing was incomplete across some antimicrobial agents, additional resistance combinations may not have been captured.

### 3.8. Resistance Stratified by Prior Eradication Therapy

A history of prior *H. pylori* eradication therapy was significantly associated with antibiotic resistance. Among patients with previous eradication therapy (*n* = 38), resistance to at least one antimicrobial agent was observed more frequently compared with treatment-naïve patients (63.2% vs. 39.2%, *p* = 0.006).

Clarithromycin resistance was more common among previously treated patients (60.5% vs. 34.6%, *p* = 0.006), as was MDR (28.9% vs. 7.7%, *p* = 0.001). Metronidazole resistance was also numerically higher among previously treated patients (47.4% vs. 31.5%, *p* = 0.071). Levofloxacin resistance was observed more frequently in patients with prior therapy (21.1% vs. 10.0%, *p* = 0.06), although this difference did not reach statistical significance. No statistically significant differences were observed for amoxicillin resistance.

These findings indicate that prior eradication therapy is strongly associated with both single-drug and MDR and contributes substantially to the resistance burden within the cohort (Table 8).

### 3.9. Predictors of Antibiotic Resistance

Multivariable logistic regression analysis was performed among the 168 patients who underwent antibiotic susceptibility testing. Resistance to at least one antimicrobial agent (*n* = 75, 44.6%) was defined as the dependent variable, representing a composite clinically relevant outcome reflecting the overall likelihood of antimicrobial resistance.

Independent variables included age (continuous), sex, place of residence (urban vs. rural), and history of prior *H. pylori* eradication therapy. Seventy-five outcome events were included, yielding an EPV ratio of 18.8, indicating adequate model stability.

In multivariable analysis, prior eradication therapy was independently associated with an increased likelihood of antibiotic resistance (aOR: 2.41; 95% CI: 1.29–4.51; *p* = 0.006). Age, sex, and place of residence were not significantly associated with resistance.

The model demonstrated acceptable discrimination, with an area under the AUC of 0.74. Calibration assessed using the Hosmer–Lemeshow goodness-of-fit test indicated good model fit (χ^2^ = 6.12, *p* = 0.63).

These findings suggest that prior eradication therapy is the principal clinical predictor of antimicrobial resistance in this cohort (Table 9).

### 3.10. Eradication Outcomes According to Treatment Strategy and Resistance Status

Eradication outcomes were available for 300 patients who underwent post-treatment confirmation using stool antigen testing or urea breath testing performed at least four weeks after completion of therapy. Overall, eradication was achieved in 228 patients (76.0%).

Cure rates were significantly higher among patients receiving susceptibility-guided therapy compared with empirical therapy. Among patients with available susceptibility data, eradication rates were lower in those with antimicrobial resistance, particularly in cases of clarithromycin resistance and MDR (Table 10).

## 4. Discussion

This study provides a comprehensive evaluation of antimicrobial resistance patterns in *H. pylori* infection and identifies clinical predictors of resistance in a real-world cohort of patients managed either with susceptibility-guided therapy or empirical eradication. Overall, the findings support the major working hypotheses, demonstrating a high burden of clarithromycin resistance, a measurable prevalence of MDR, and a strong independent association between prior eradication therapy and antimicrobial resistance.

### 4.1. Resistance Burden and Distribution of Susceptibility Patterns

A principal finding of this study is the substantial prevalence of antimicrobial resistance among patients who underwent susceptibility testing, with resistance to at least one agent detected in 44.6% of patients with available susceptibility data. Clarithromycin resistance was the most frequent pattern (42.5%, 68/160 tested isolates), followed by metronidazole resistance (36.4%, 59/162), while levofloxacin resistance was lower (14.0%, 21/150). In contrast, amoxicillin resistance remained uncommon (2.4%, 4/164), rifampicin resistance was rare (1.7%, 2/120).

Overall, this susceptibility profile is consistent with international surveillance reports showing that resistance to clarithromycin and metronidazole remains widespread and represents a major contributor to declining eradication success with commonly used empirical regimens [27,28,29,30]. Conversely, the low prevalence of amoxicillin resistance observed in our cohort aligns with most contemporary datasets and supports the continued role of amoxicillin as a backbone component of first-line and rescue therapies, including susceptibility-guided approaches [31,32,33,34,35].

### 4.2. MDR and Clinically Relevant Resistance Combinations

MDR (defined as resistance to ≥2 antimicrobial classes) was identified in 12.5% of patients in the susceptibility-tested cohort. Among isolates with available susceptibility data, dual resistance to clarithromycin and metronidazole was the most commonly observed MDR pattern. However, interpretation of MDR distribution should be made cautiously because not all isolates underwent susceptibility testing against all antimicrobial agents. Consequently, incomplete testing coverage may have limited the detection of less frequent or more complex resistance combinations.

Despite these limitations, the observed frequency of clarithromycin–metronidazole dual resistance remains clinically important. Resistance involving these two antimicrobial classes may directly compromise the effectiveness of standard clarithromycin-based triple therapy and may also reduce the efficacy of non-bismuth concomitant or sequential regimens in which both agents are key therapeutic components. Previous studies have similarly identified clarithromycin–metronidazole dual resistance as an important contributor to treatment failure and reduced eradication success [36,37,38,39,40].

Accordingly, although the precise distribution of MDR patterns cannot be definitively characterized in the present dataset, the findings support the clinical relevance of combined resistance to clarithromycin and metronidazole and underscore the importance of resistance-informed treatment selection in high-resistance settings.

### 4.3. Prior Eradication Therapy as the Main Driver of Resistance

The most important finding of this study is the independent association between prior *H. pylori* eradication therapy and antimicrobial resistance. Patients with previous treatment exposure exhibited significantly higher rates of resistance to at least one antibiotic, clarithromycin resistance, and MDR compared with treatment-naïve individuals. In multivariable logistic regression analysis, prior eradication therapy remained the only statistically significant independent predictor of resistance (aOR 2.41; 95% CI 1.29–4.51; *p* = 0.006), whereas age, sex, and place of residence were not associated with resistance risk.

This observation is biologically plausible and consistent with existing evidence demonstrating that repeated antibiotic exposure promotes selection of resistant *H. pylori* strains and contributes to secondary resistance following treatment failure. Macrolide-containing regimens are known to select for point mutations in the 23S rRNA gene that confer clarithromycin resistance [33,35,41]. The markedly higher prevalence of MDR among previously treated patients in our cohort underscores the cumulative effect of antimicrobial pressure and highlights prior eradication therapy as a clinically meaningful marker of increased resistance risk.

Collectively, these findings emphasize the importance of systematically documenting treatment history and avoiding reuse of previously administered antibiotic combinations, particularly clarithromycin-based regimens, in patients with prior eradication attempts [33,42].

### 4.4. Implications for Empirical Therapy and Antimicrobial Stewardship

The resistance patterns observed in this study have important implications for empirical treatment strategies and antimicrobial stewardship in *H. pylori* infection. Most notably, the clarithromycin resistance rate of 42.5% substantially exceeds the 15–20% threshold above which empirical clarithromycin-based triple therapy is generally discouraged according to international consensus guidelines [13,43,44]. In this context, continued routine use of clarithromycin-containing regimens is likely to result in suboptimal eradication rates and increased need for retreatment, particularly among patients with prior therapy exposure.

Given the high prevalence of clarithromycin and metronidazole resistance, alternative empirical regimens—such as bismuth-containing quadruple therapy—may represent more appropriate first-line options in this setting [45,46,47,48]. Where available, antibiotic susceptibility testing can further optimize regimen selection by enabling individualized therapy, especially in patients with previous eradication failure or known macrolide exposure.

From an antimicrobial stewardship perspective, these findings underscore the need to minimize unnecessary use of macrolides and fluoroquinolones, both within eradication protocols and in other clinical indications, to reduce selective pressure for resistance development [49,50,51,52]. Systematic assessment of prior antibiotic exposure, adherence to guideline-recommended regimens, and avoidance of repeated empirical reuse of the same antibiotic classes are essential components of responsible stewardship.

Overall, these findings support consideration of a shift away from uniform empirical strategies toward more rational, resistance-informed approaches in similar high-resistance settings.

These recommendations should be interpreted within the context of a single-center tertiary-care setting with a high prevalence of antimicrobial resistance and may not be directly generalizable to regions with different resistance patterns or healthcare structures.

### 4.5. Strengths and Limitations

This study has several notable strengths. It includes a relatively large and well-characterized clinical cohort of patients with confirmed *H. pylori* infection, enabling a detailed assessment of antimicrobial susceptibility patterns across multiple antibiotic classes. The systematic evaluation of resistance in the study group provides clinically relevant real-world susceptibility data despite the limitations related to incomplete testing coverage. Furthermore, the multivariable logistic regression analysis was conducted with an adequate events-per-variable ratio, ensuring statistical stability. The predictive model demonstrated acceptable discrimination (AUC = 0.74) and good calibration, supporting the robustness of the conclusion that prior eradication therapy is an independent predictor of antimicrobial resistance.

Nevertheless, several limitations should be considered. First, susceptibility testing was performed only in the study group, restricting resistance analyses to a subset of the overall cohort and potentially introducing selection bias, particularly if testing was more frequently performed in patients with prior treatment or more complex clinical presentations. Second, due to laboratory constraints, not all isolates were tested against every antimicrobial agent, resulting in variable denominators across antibiotics (ranging from 120 to 164 tested isolates). This incomplete susceptibility testing represents an important methodological limitation because the missing susceptibility data may have introduced additional uncertainty regarding the completeness of resistance characterization. Consequently, selection bias cannot be excluded, particularly if isolates undergoing more comprehensive testing differed systematically from those with incomplete susceptibility profiles.

This limitation may affect the precision, comparability, and completeness of resistance estimates across antimicrobial agents and may have reduced the ability to identify less common or more complex MDR combinations. Therefore, the reported MDR distribution should be interpreted cautiously and considered exploratory rather than definitive, as incomplete susceptibility coverage may have influenced the observed resistance patterns. Additionally, biopsy sampling was not fully standardized and was guided by clinical judgment, which may introduce variability in microbiological yield, although no significant differences in biopsy site distribution were observed between groups. In addition, the non-randomized allocation of patients to study groups introduces the potential for indication bias, as group assignment was influenced by clinical judgment and logistical considerations. Although multivariable regression analysis was used to adjust for measured confounders, residual confounding cannot be excluded. In addition, although age differed modestly between groups, it was not identified as an independent predictor of antimicrobial resistance, suggesting limited impact on the primary outcome. Propensity score-based methods were not applied due to the limited sample size and number of events, which could compromise model stability and reliability, but this represents an important consideration for future studies.

Furthermore, multivariable analysis was performed using a composite outcome defined as resistance to at least one antimicrobial agent. While this approach provides a clinically relevant measure of overall resistance risk, it does not allow identification of predictors specific to individual antibiotics. Antibiotic-specific regression models were not performed due to variable testing coverage across antimicrobial agents and limited numbers of resistance events for certain drugs, which could compromise the statistical robustness of the models.

Third, information regarding prior eradication therapy relied on available clinical documentation and patient history, and detailed data on adherence, antibiotic dosing, and treatment duration were not consistently captured. These factors may influence the development of secondary resistance and could not be fully accounted for in the analysis. Fourth, molecular mechanisms of resistance—such as 23S rRNA mutations associated with clarithromycin resistance or gyrA mutations linked to fluoroquinolone resistance—were not evaluated, limiting mechanistic interpretation and preventing genotype–phenotype correlation.

Additionally, eradication outcomes were available only for a subset of patients with documented post-treatment follow-up, reflecting the retrospective design and variability in clinical practice. As a result, analyses of treatment success were conducted using an available-case approach, which may introduce selection bias if patients with available follow-up differ systematically from those without documented test-of-cure results. Furthermore, eradication outcomes were not predefined as a primary endpoint of the study, and therefore the analyses should be considered exploratory. Consequently, while the observed associations between antimicrobial resistance and treatment outcomes are clinically meaningful and consistent with existing evidence, they should be interpreted with caution.

Finally, the single-center design in a tertiary care setting may limit the generalizability of these findings, as such centers may include a higher proportion of patients with more complex clinical presentations, prior treatment exposure, or refractory infection. This may lead to an overrepresentation of cases with increased antimicrobial resistance compared with the general population. Multicenter studies with standardized and more complete susceptibility testing across antimicrobial agents are needed to better characterize MDR patterns and validate the resistance distributions observed in the present study.

### 4.6. Future Directions

The findings of this study highlight several priorities for future research and clinical development.

First, multicenter and longitudinal surveillance studies are needed to monitor regional and temporal trends in *H. pylori* antimicrobial resistance. Given the dynamic nature of antibiotic prescribing practices and resistance evolution, continuous epidemiological monitoring is essential to inform guideline adaptation and empirical treatment recommendations.

Second, integration of molecular diagnostic techniques into routine practice may enhance resistance detection. Identification of 23S rRNA mutations associated with clarithromycin resistance and gyrA mutations linked to fluoroquinolone resistance could enable faster, less invasive resistance profiling and support more timely individualized treatment selection. Combining phenotypic susceptibility testing with molecular methods may provide a more comprehensive understanding of local resistance ecology.

Third, prospective comparative studies evaluating susceptibility-guided or resistance-informed approaches may be particularly beneficial in comparable high-resistance contexts. Such studies should assess not only eradication success rates but also adverse events, cost-effectiveness, and long-term outcomes, including recurrence and reinfection rates. These data would help determine whether broader implementation of routine susceptibility testing is clinically and economically justified.

Finally, future research should explore antimicrobial stewardship-informed strategies that incorporate prior antibiotic exposure, eradication history, and regional resistance patterns into individualized decision-making algorithms. Development of risk-based treatment pathways and predictive models may improve first-line eradication success while minimizing repeated treatment failures and the amplification of resistance. Future research should also incorporate antibiotic-specific predictive modeling to better identify clinical determinants of resistance to individual agents and support more tailored therapeutic strategies.

Collectively, these directions underscore the need for a more personalized and resistance-informed approach to *H. pylori* management, integrating epidemiological surveillance, molecular diagnostics, and stewardship principles to optimize therapeutic outcomes.

In high-resistance settings such as ours, reliance on empirical clarithromycin-based therapy is increasingly untenable, and susceptibility-guided or resistance-informed treatment strategies should be prioritized.

## 5. Conclusions

This study demonstrates a high prevalence of clarithromycin resistance among patients with *H. pylori* infection in our setting, while amoxicillin resistance remained uncommon. Among isolates with available susceptibility data, dual resistance involving clarithromycin and metronidazole was the most frequently observed MDR pattern. However, incomplete susceptibility testing across antimicrobial agents limits definitive characterization of the full MDR distribution and warrants cautious interpretation of these findings.

Prior eradication therapy emerged as the principal independent predictor of antimicrobial resistance, underscoring the critical importance of detailed treatment history when selecting subsequent regimens. Demographic factors were not significantly associated with resistance risk.

Given that clarithromycin resistance substantially exceeds internationally accepted thresholds for empirical triple therapy, reliance on uniform clarithromycin-based regimens may be suboptimal in this setting. Greater consideration should be given to susceptibility-guided strategies or alternative empirical regimens aligned with contemporary resistance patterns.

Overall, these findings support the importance of resistance-informed and stewardship-oriented management approaches to optimize eradication success while highlighting the need for further studies with standardized and complete susceptibility testing to better define MDR patterns in *H. pylori* infection.

## Figures and Tables

**Table 1:** Demographic Characteristics of the Study Population (*n* = 352).

Parameter	Study Group (*n* = 168)	Control Group (*n* = 184)	*p*-Value	Total (*n* = 352)
Age (years), mean ± SD	49.6 ± 15.2	53.9 ± 15.7	0.012	51.8 ± 15.6
Male sex, *n* (%)	98 (58.3)	92 (50.0)	0.118	190 (54.0)
Urban origin, *n* (%)	104 (61.9)	109 (59.2)	0.642	213 (60.5)

**Table 2:** Clinical symptoms in the study and control groups (*n* = 352).

Symptom	Study Group (*n* = 168)	Control Group (*n* = 184)	*p*-Value
Melena, *n* (%)	19 (11.3)	11 (6.0)	0.082
Diffuse abdominal pain, *n* (%)	108 (64.3)	108 (58.7)	0.284
Epigastric pain, *n* (%)	155 (92.3)	169 (91.8)	0.861
Nausea, *n* (%)	55 (32.7)	67 (36.4)	0.457
Flatulence, *n* (%)	118 (70.2)	132 (71.7)	0.756
Hematemesis, *n* (%)	6 (3.6)	9 (4.9)	0.541
Loss of appetite, *n* (%)	84 (50.0)	99 (53.8)	0.488
Rectal bleeding, *n* (%)	5 (3.0)	7 (3.8)	0.674
Vomiting, *n* (%)	18 (10.7)	22 (12.0)	0.710
Heartburn, *n* (%)	27 (16.1)	31 (16.8)	0.872

**Table 3:** History of previous *H. pylori* eradication therapy (*n* = 352).

Previous Eradication Regimen	Study Group (*n* = 168)	Control Group (*n* = 184)	*p*-Value
PPI + amoxicillin + clarithromycin	21 (12.5%)	14 (7.6%)	—
PPI + amoxicillin + clarithromycin ± levofloxacin	9 (5.4%)	4 (2.2%)	—
PPI + amoxicillin + levofloxacin	8 (4.8%)	3 (1.6%)	—
Any previous eradication therapy (total)	38 (22.6%)	21 (11.4%)	0.006

Note: Abbreviations: PPI (proton pump inhibitor). — indicates not applicable.

**Table 4:** Endoscopic findings in the study and control groups (*n* = 352).

Lesion/Pathology	Study Group (*n* = 168)	Control Group (*n* = 184)	*p*-Value
Gastritis			
No visible lesion, *n* (%)	10 (6.0)	13 (7.1)	0.674
Erythematous gastritis, *n* (%)	78 (46.4)	90 (48.9)	0.648
Erosive gastritis, *n* (%)	31 (18.5)	30 (16.3)	0.583
Edematous gastritis, *n* (%)	20 (11.9)	22 (12.0)	0.981
Atrophic gastritis, *n* (%)	15 (8.9)	19 (10.3)	0.671
Ulcer disease			
Esophageal ulcerations, *n* (%)	4 (2.4)	5 (2.7)	1.000 †
Gastric ulcers, *n* (%)	16 (9.5)	12 (6.5)	0.284
Duodenal ulcers, *n* (%)	19 (11.3)	16 (8.7)	0.411
Gastric cancer, *n* (%)	0 (0.0)	0 (0.0)	—

Note: *p*-values were calculated using the Chi-square test; † Fisher’s exact test was used when expected cell counts were <5 in any cell. — indicates not applicable.

**Table 5:** Antibiotic susceptibility patterns in the study group (*n* = 168).

Antibiotic	Tested (*n*)	Susceptible, *n* (%)	Resistant, *n* (%)
Amoxicillin	164	160 (97.6)	4 (2.4)
Clarithromycin	160	92 (57.5)	68 (42.5)
Metronidazole	162	103 (63.6)	59 (36.4)
Levofloxacin	150	129 (86.0)	21 (14.0)
Rifampicin	120	118 (98.3)	2 (1.7)

**Table 6:** Distribution of resistance patterns in the study group (*n* = 168).

Resistance Category	*n* (%)
Fully susceptible (no resistance)	93 (55.4)
Single-drug resistance	54 (32.1)
Multidrug resistance (≥2 antimicrobial classes)	21 (12.5)
Dual resistance (clarithromycin + metronidazole)	21 (12.5)

Note: Resistance categories are based on available susceptibility data for each isolate. Because not all isolates were tested against all antimicrobial agents, incomplete susceptibility profiles may have limited identification of additional or more complex MDR patterns.

**Table 7:** Observed dual resistance patterns based on available susceptibility data.

Resistance Combination	*n* (%)
Clarithromycin + Metronidazole	21 (12.5)
Clarithromycin + Levofloxacin	0 (0.0)
Metronidazole + Levofloxacin	0 (0.0)

Note: Resistance combinations are reported based on available susceptibility data. Because not all isolates underwent testing against all antimicrobial agents, the absence of additional resistance combinations should not be interpreted as definitive evidence that such patterns were not present in the study population.

**Table 8:** Antibiotic Resistance According to Prior Eradication Therapy (*n* = 168).

Resistance Pattern	Prior Therapy (*n* = 38)	No Prior Therapy (*n* = 130)	*p*-Value *
Any resistance (≥1 drug)	24 (63.2)	51 (39.2)	0.006
Clarithromycin resistance	23 (60.5)	45 (34.6)	0.006
Metronidazole resistance	18 (47.4)	41 (31.5)	0.071
Levofloxacin resistance	8 (21.1)	13 (10.0)	0.09
Amoxicillin resistance	2 (5.3)	2 (1.5)	0.214 †
Multidrug resistance (≥2 antimicrobial classes)	11 (28.9)	10 (7.7)	0.001

* *p*-values were calculated using the Chi-square test; † Fisher’s exact test was used due to expected cell counts < 5. Percentages are calculated based on the total number of patients within each subgroup.

**Table 9:** Multivariable logistic regression analysis for predictors of antibiotic resistance (*n* = 168).

Predictor	Adjusted OR	95% CI	*p*-Value
Age (per year increase)	1.01	0.99–1.03	0.248
Male sex	1.18	0.64–2.17	0.592
Urban residence	0.93	0.51–1.71	0.817
Prior eradication therapy	2.41	1.29–4.51	0.006

Note: Outcome variable: resistance to at least one antimicrobial agent (*n* = 75 events, 44.6%). Model performance: area under the AUC = 0.74. Calibration assessed using the Hosmer–Lemeshow goodness-of-fit test (χ^2^ = 6.12, *p* = 0.63). EPV = 18.8.

**Table 10:** Eradication outcomes according to treatment strategy and resistance status (*n* = 300).

Variable	Patients With Follow-Up, *n*	Eradicated, *n* (%)	Not Eradicated, *n* (%)	*p*-Value
Overall cohort	300	228 (76.0)	72 (24.0)	—
Treatment strategy				
Susceptibility-guided therapy	145	122 (84.1)	23 (15.9)	0.001
Empirical therapy	155	106 (68.4)	49 (31.6)	
Resistance status (tested subgroup)				
No resistance detected	82	74 (90.2)	8 (9.8)	<0.001
Any resistance detected	63	48 (76.2)	15 (23.8)	
Clarithromycin resistance				
Clarithromycin susceptible	82	74 (90.2)	8 (9.8)	<0.001
Clarithromycin resistant	63	38 (60.3)	25 (39.7)	
Multidrug resistance				
No Multidrug resistance	127	112 (88.2)	15 (11.8)	0.004
Multidrug resistance	18	10 (55.6)	8 (44.4)	

## Data Availability

The original contributions presented in this study are included in the article. Further inquiries can be directed to the corresponding authors. Access is subject to institutional approval and privacy regulations due to the use of clinical patient data.

## Abbreviations

The following abbreviations are used in this manuscript:

AUC	Area Under the Receiver Operating Characteristic Curve
CI	Confidence Interval
EPV	Events Per Variable
MDR	Multidrug Resistance
OR	Odds Ratio
aOR	Adjusted Odds Ratio
PPI	Proton Pump Inhibitor
RUT	Rapid Urease Test

## References

[1] Li Y., Choi H., Leung K., Jiang F., Graham D.Y., Leung W.K. (2023). Global prevalence of Helicobacter pylori infection between 1980 and 2022: A systematic review and meta-analysis. Lancet Gastroenterol. Hepatol..

[2] Namikawa K., Purisevic F.L., Thorsteinsson J.B., Bjornsson E.S. (2025). Helicobacter pylori across continents: Contrasts in epidemiology, genetics, clinical impact, and management between East and West. Int. J. Mol. Sci..

[3] Andreev D.N., Khurmatullina A.R., Maev I.V., Bordin D.S., Abdulkhakov S.R., Kucheryavyy Y.A., Beliy P.A., Sokolov F.S. (2025). The prevalence of Helicobacter pylori infection in the adult population of Russia: A systematic review and meta-analysis. Epidemiologia.

[4] Reyes V.E. (2023). Helicobacter pylori and its role in gastric cancer. Microorganisms.

[5] Duan Y., Xu Y., Dou Y., Xu D. (2025). Helicobacter pylori and gastric cancer: Mechanisms and new perspectives. J. Hematol. Oncol..

[6] Kinpelu O.O., Samuel H.S., Undie D.A., Ibekwe F.A., Onotu O.P., Etim E.E. (2025). Current concepts of Helicobacter pylori infection. Eur. J. Med. Res..

[7] Myrou A. (2024). Molecular mechanisms and treatment strategies for Helicobacter pylori-induced gastric carcinogenesis and mucosa-associated lymphoid tissue (MALT) lymphoma. Cureus.

[8] Chey W.D., Howden C.W., Moss S.F., Morgan D.R., Greer K.B., Grover S., Shah S.C. (2024). ACG clinical guideline: Treatment of Helicobacter pylori infection. Am. J. Gastroenterol..

[9] Malfertheiner P., Megraud F., Rokkas T., Gisbert J.P., Liou J.M., Schulz C., Gasbarrini A., Hunt R.H., Leja M., O’Morain C. (2022). Management of Helicobacter pylori infection: The Maastricht VI/Florence consensus report. Gut.

[10] Farsetti A., Illi B., Gaetano C. (2023). How epigenetics impacts on human diseases. Eur. J. Intern. Med..

[11] Catarci M., Guadagni S., Masedu F., Sartelli M., Montemurro L.A., Baiocchi G.L., Tebala G.D., Borghi F., Marini P., Scatizzi M. (2024). Oral antibiotics alone versus oral antibiotics combined with mechanical bowel preparation for elective colorectal surgery: A propensity score-matching re-analysis of the iCral 2 and 3 prospective cohorts. Antibiotics.

[12] Matei S.D., Suciu R.N., Ilias T., Hocopan C., Frățilă O. (2025). Global Dynamics of Research on Antibiotic Resistance in *Helicobacter pylori*: A Bibliometric Analysis. Gastrointest. Disord..

[13] Schulz C., Liou J.M., Alboraie M., Bornschein J., Campos Nunez C., Coelho L.G., Quach D.T., Fallone C.A., Chen Y.C., Gerhard M. (2025). Helicobacter pylori antibiotic resistance: A global challenge in search of solutions. Gut.

[14] Jung H.K., Kang S.J., Lee Y.C., Yang H.J., Park S.Y., Shin C.M., Kim S.E., Lim H.C., Kim J.H., Nam S.Y. (2021). Evidence-based guidelines for the treatment of Helicobacter pylori infection in Korea 2020. Gut Liver.

[15] Cho J.-H., Jin S.-Y. (2025). Efficacy and safety of modified bismuth quadruple therapy for first-line Helicobacter pylori eradication: A systematic review and meta-analysis of randomized controlled trials. Microorganisms.

[16] Katelaris P., Hunt R., Bazzoli F., Cohen H., Fock K.M., Gemilyan M., Malfertheiner P., Mégraud F., Piscoya A., Quach D. (2023). Helicobacter pylori World Gastroenterology Organization global guideline. J. Clin. Gastroenterol..

[17] Nyssen O.P., Espada M., Gisbert J.P. (2022). Empirical vs. susceptibility-guided treatment of Helicobacter pylori infection: A systematic review and meta-analysis. Front. Microbiol..

[18] Francesco V., Zullo A., Manta R., Satriano A., Fiorini G., Pavoni M., Saracino I.M., Giostra F., Monti G., Vaira D. (2022). Culture-based antibiotic susceptibility testing for Helicobacter pylori infection: A systematic review. Ann. Gastroenterol..

[19] Ng H.-Y., Leung W.K., Cheung K.-S. (2023). Antibiotic resistance, susceptibility testing and stewardship in Helicobacter pylori infection. Int. J. Mol. Sci..

[20] Sohn J.Y., Bang C.S., Choi A.I., Choi J.-G., Gong E.J. (2025). Antimicrobial resistance patterns and determinants of Helicobacter pylori culture success: A prospective study. Antibiotics.

[21] Shao Y., Lin Y., Fang Z., Yan J., Zheng T., Ye G. (2024). Analysis of Helicobacter pylori resistance in patients with different gastric diseases. Sci. Rep..

[22] Salahi-Niri A., Nabavi-Rad A., Monaghan T.M., Rokkas T., Doulberis M., Sadeghi A., Zali M.R., Yamaoka Y., Tacconelli E., Yadegar A. (2024). Global prevalence of Helicobacter pylori antibiotic resistance among children in the World Health Organization regions between 2000 and 2023: A systematic review and meta-analysis. BMC Med..

[23] Osser G., Osser B., Toth C., Miuța C.C., Marconi G.R., Bondar L.I. (2024). Exploring the relationship between ejection fraction, arterial stiffness, NT-proBNP, and hospitalization risk in heart failure patients. Diagnostics.

[24] Megraud F., Bruyndonckx R., Coenen S., Wittkop L., Huang T.D., Hoebeke M., Bénéjat L., Lehours P., Goossens H., Glupczynski Y. (2021). Helicobacter pylori resistance to antibiotics in Europe in 2018 and its relationship to antibiotic consumption in the community. Gut.

[25] Mégraud F., Graham D.Y., Howden C.W., Trevino E., Weissfeld A., Hunt B., Smith N., Leifke E., Chey W.D. (2023). Rates of antimicrobial resistance in Helicobacter pylori isolates from clinical trial patients across the US and Europe. Am. J. Gastroenterol..

[26] Osser B., Toth C., Nistor-Cseppento C.D., Osser G., Miuța C.C., Ilia I., Iovanovici D.C., Aur C., Bondar L.I. (2025). Evaluating tech neck: A pilot study using a self-developed questionnaire on symptoms, posture, and preventive measures. Children.

[27] Dossouvi K.M., Bouyo T., Sognonnou S., Ibadin E.E., Lv L.C., Sambe Ba B., Seck A., Dossim S., Sellera F.P., Camara M. (2025). Clarithromycin-resistant Helicobacter pylori in Africa: A systematic review and meta-analysis. Antimicrob. Resist. Infect. Control.

[28] Alba C., Blanco A., Alarcón T. (2017). Antibiotic resistance in Helicobacter pylori. Curr. Opin. Infect. Dis..

[29] Mladenova I. (2023). Epidemiology of Helicobacter pylori resistance to antibiotics (a narrative review). Antibiotics.

[30] Osser B., Toth C., Nistor-Cseppento C.D., Cevei M., Aur C., Orodan M., Fazakas R., Bondar L.I. (2025). Predictors of problematic internet use among Romanian high school students. Children.

[31] Liu L., Nahata M.C. (2024). Newer therapies for refractory Helicobacter pylori infection in adults: A systematic review. Antibiotics.

[32] Nyssen O.P., Bordin D., Tepes B., Pérez-Aisa Á., Vaira D., Caldas M., Bujanda L., Castro-Fernandez M., Lerang F., Leja M. (2021). European registry on Helicobacter pylori management (Hp-EuReg): Patterns and trends in first-line empirical eradication prescription and outcomes of 5 years and 21,533 patients. Gut.

[33] Bujanda L., Nyssen O.P., Vaira D., Saracino I.M., Fiorini G., Lerang F., Georgopoulos S., Tepes B., Heluwaert F., Gasbarrini A. (2021). Antibiotic resistance prevalence and trends in patients infected with Helicobacter pylori in the period 2013–2020: Results of the European registry on H. pylori management (Hp-EuReg). Antibiotics.

[34] Ho J.J.C., Navarro M., Sawyer K., Elfanagely Y., Moss S.F. (2022). Helicobacter pylori antibiotic resistance in the United States between 2011 and 2021: A systematic review and meta-analysis. Am. J. Gastroenterol..

[35] Yu Y., Xue J., Lin F., Liu D., Zhang W., Ru S., Jiang F. (2024). Global primary antibiotic resistance rate of Helicobacter pylori in recent 10 years: A systematic review and meta-analysis. Helicobacter.

[36] Hwang J.Y., Kim C., Kwon Y.H., Lee J.E., Jeon S.W., Nam S.Y., Seo A.N., Han M.H., Park J.H. (2021). Dual clarithromycin and metronidazole resistance is the main cause of failure in ultimate Helicobacter pylori eradication. Dig. Dis..

[37] Khalid A., Tabish S., Burhan M., Saad M., Gul I., Abid H.M.F., Hanif M.S., Abdul Samad Z., Rasool A., Daniyal S.M. (2025). Concomitant versus tailored therapy based on antibiotic resistance profiles for Helicobacter pylori eradication: A systematic review and meta-analysis. JGH Open.

[38] Losurdo G., Pricci M., De Bellis M., Celiberto F., Russo F., Riezzo G., D’attoma B., Iannone A., Rendina M., Ierardi E. (2022). Effect of metronidazole resistance on Helicobacter pylori eradication regimens. J. Dig. Dis..

[39] Parra P., Nyssen O.P., Gisbert J.P. (2026). Non-bismuth quadruple concomitant treatment for Helicobacter pylori eradication: A systematic review and meta-analysis. Helicobacter.

[40] Hasanuzzaman M., Bang C.S., Gong E.J. (2024). Antibiotic resistance of Helicobacter pylori: Mechanisms and clinical implications. J. Korean Med. Sci..

[41] Yan Z., Huang B., Yang K., Anaman R., Amanze C., Jin J., Zhou H., Qiu G., Zeng W. (2023). Enlarging the substrate binding pocket of penicillin G acylase from Achromobacter sp. for highly efficient biosynthesis of β-lactam antibiotics. Bioorg. Chem..

[42] Xue Z., Zhao Q., Pei F., Gong Y., Wang F., Wang Y., Chen Q., Li Y., Xu Q., Tian J. (2025). Helicobacter pylori antimicrobial resistance and gene variants in Shandong Province. Sci. Rep..

[43] Elbehiry A., Abalkhail A., Anajirih N., Alkhamisi F., Aldamegh M., Alramzi A., AlShaqi R., Alotaibi N., Aljuaid A., Alzahrani H. (2024). Helicobacter pylori: Routes of infection, antimicrobial resistance, and alternative therapies as a means to develop infection control. Diseases.

[44] Malfertheiner P., Megraud F., Rokkas T., Gisbert J.P. (2024). Empiric use of standard triple therapy in Helicobacter pylori eradication does not require readjustment in the clarithromycin resistance cut-off point. Gut.

[45] Veldhuyzen van Zanten S., Krahn T. (2025). Do the new ACG Helicobacter pylori treatment guidelines have implications for Canada?. J. Can. Assoc. Gastroenterol..

[46] Alcedo J., Gracia M., García-Cámara P., Palacín C., Gallego S., Jimeno-Ayllon C., Frago S., Aured I., Luzón L. (2020). Prospective comparative study between two first-line regimens for Helicobacter pylori eradication: Non-bismuth quadruple versus bismuth quadruple therapy. Gastroenterol. Hepatol..

[47] Gisbert J.P., Parra P., Nyssen O.P. (2026). Review article: Classic bismuth quadruple therapy for Helicobacter pylori infection—Questions focused on clinical practice. Aliment. Pharmacol. Ther..

[48] Osser B., Toth C., Osser G., Nistor-Cseppento C.D., Niculescu V., Bondar L.I. (2025). Student behavior and its association with multi-device addiction and back pain in Western Romania. Balneo PRM Res. J..

[49] Rocha G.R., Lemos F.F.B., Silva L.G.O., Luz M.S., Correa Santos G.L., Rocha Pinheiro S.L., Calmon M.S., de Melo F.F. (2025). Overcoming antibiotic-resistant Helicobacter pylori infection: Current challenges and emerging approaches. World J. Gastroenterol..

[50] Olivieri R., Vannini P., Corzani A., Bianco M.T., Franchi F., Cusi M.G., Scolletta S., Arena F., Basagni C., Gusinu R. (2023). Rapid decrease in fluoroquinolones consumption following implementation of a simple antimicrobial stewardship bundled intervention in a university hospital during the COVID-19 pandemic. Antibiotics.

[51] Morad Kasani S., Mofid M., Navidifar T., Golab N., Parvizi E., Badmasti F., Sholeh M., Beig M. (2024). Insights into Helicobacter pylori macrolide resistance: A comprehensive systematic review and meta-analysis. Front. Microbiol..

[52] Elbaiomy R.G., Luo X., Guo R., Deng S., Du M., El-Sappah A.H., Bakeer M., Azzam M.M., Elolimy A.A., Madkour M. (2025). Antibiotic resistance in Helicobacter pylori: A genetic and physiological perspective. Gut Pathog..

